# Blockchain-Based Peer-to-Peer Transactive Energy Management Scheme for Smart Grid System

**DOI:** 10.3390/s22134826

**Published:** 2022-06-26

**Authors:** Aparna Kumari, Urvi Chintukumar Sukharamwala, Sudeep Tanwar, Maria Simona Raboaca, Fayez Alqahtani, Amr Tolba, Ravi Sharma, Ioan Aschilean, Traian Candin Mihaltan

**Affiliations:** 1 Department of Computer Science and Engineering, Institute of Technology, Nirma University, Ahmedabad 382481, Gujarat, India; aparna.kumari@nirmauni.ac.in; 2 Computer Science and Engineering Department, R. N. G. Patel Institute of Technology, Bardoli, Surat 394620, Gujarat, India; urvi.sukharamwala19@gmail.com; 3 National Research and Development Institute for Cryogenic and Isotopic Technologies—ICSI Rm. Valcea, Uz-inei Street, No. 4, P.O. Box 7 Raureni, 240050 Ramnicu Valcea, Romania; simona.raboaca@icsi.ro; 4 Software Engineering Department, College of Computer and Information Sciences, King Saud University, Riyadh 12372, Saudi Arabia; fhalqahtani@ksu.edu.sa; 5 Computer Science Department, Community College, King Saud University, Riyadh 11437, Saudi Arabia; atolba@ksu.edu.sa; 6 Centre for Inter-Disciplinary Research and Innovation, University of Petroleum and Energy Studies, Dehradun 248007, Uttarakhand, India; ravisharmacidri@gmail.com; 7 Ioan Aşchilean Faculty of Civil Engineering, Civil Engineering and Management Department & Research, Technological Development and Innovation Centre in Civil and Building Services Engineering, Technical University of Cluj—Napoca, C-tin Daicoviciu Street, No. 15, 400020 Cluj-Napoca, Romania; 8 Traian Candin Mihălţan Faculty of Building Services Engineering, Technical University of Cluj—Napoca, Bd. 21 Decembrie 1989, No. 128-130, 400604 Cluj-Napoca, Romania; mihaltantraian@yahoo.com

**Keywords:** blockchain, smart grid, transactive energy management, peer-to-peer energy trading, ethereum

## Abstract

In Smart Grid (SG), Transactive Energy Management (TEM) is one of the most promising approaches to boost consumer participation in energy generation, energy management, and establishing decentralized energy market models using Peer-to-Peer (P2P). In P2P, a prosumer produces electric energy at their place using Renewable Energy Resources (RES) such as solar energy, wind energy, etc. Then, this generated energy is traded with consumers (who need the energy) in a nearby locality. P2P facilitates energy exchange in localized micro-energy markets of the TEM system. Such decentralized P2P energy management could cater to diverse prosumers and utility business models. However, the existing P2P approaches suffer from several issues such as single-point-of-failure, network bandwidth, scalability, trust, and security issues. To handle the aforementioned issues, this paper proposes a Decentralized and Transparent P2P Energy Trading *(DT-P2PET)* scheme using blockchain. The proposed *DT-P2PET* scheme aims to reduce the grid’s energy generation and management burden while also increasing profit for both consumers and prosumers through a dynamic pricing mechanism. The *DT-P2PET* scheme uses Ethereum-blockchain-based Smart Contracts (SCs) and InterPlanetary File System (IPFS) for the P2P energy trading. Furthermore, a recommender mechanism is also introduced in this study to increase the number of prosumers. The Ethereum SCs are designed and deployed to perform P2P in real time in the proposed *DT-P2PET* scheme. The *DT-P2PET* scheme is evaluated based on the various parameters such as profit generation (for prosumer and consumer both), data storage cost, network bandwidth, and data transfer rate in contrast to the existing approaches.

## 1. Introduction

With the advancement of technologies, the penetration of rooftop Solar PhotoVoltaics (SPV) [1] and Distributed Energy Resources (DER) such as Electric Vehicles (EVs) and Battery Energy Storage Systems (BESS) has increased consumer participation in energy generation and management in the Smart Grid (SG) system [2,3,4,5]. In SG, one of the most crucial approaches, i.e., Peer-to-Peer (P2P) transactive energy trading, reduces the burden on electric utility companies. P2P increases renewable energy deployment, balances demand–response, and adds flexibility to the grid. In traditional energy supply, consumers purchase energy from retailers/utilities through time-of-use/fixed tariffs. In line with this, prosumers (who produce as well as consume or self-consumers) [6] can sell excess energy back to the SG using the *buy-back rate*. Nevertheless, tariffs for energy supply to consumers are higher than the buy-back rates for the prosumers. Here, the involvement of retailers/utility companies is required for energy trading. Furthermore, P2P establishes decentralized energy market models by generating energy from renewable energy sources such as SPV and windmills [7]. In P2P, two or more SG-connected prosumers and consumers transact energy without the involvement of grid or utility companies [8]. Numerous research work has already been done in this regard [9,10,11,12,13]. Javadi et al.  [9] analyzed a fully decentralized model for energy trading inside a Transactive Energy Management (TEM) system and presented a P2P trading framework. Consumers receive get a good offer from prosumers at a reasonable price in a decentralized system. So, P2P necessitates transparency while dealing with the consumers in a decentralized system [14].

Based on the available literature on P2P energy trading, energy markets can be categorized into four categories: (i) Full P2P market: Here, energy is traded directly between prosumers and consumers. It has scaling issues (i.e., determining a target price) related to the P2P negotiating process and the local energy balance that SG operators manage. For example, Sorin et al. [15] advocated for a comprehensive P2P network for both prosumers and consumers without the need for a centralized body. (ii) Community-based markets: Here, the SG community coordinator is responsible for negotiating prices among all prosumers and consumers to have an equilibrium price. Next, a P2P trading model for microgrid demand–response was proposed by Liu et al. utilizing a dynamic internal pricing model in which intrinsic prices are used for trading among local prosumers and consumers [10]. The price change can be influenced by a consumer’s perception of how much they are willing to pay for an item at a particular time, competing pricing, and other variables [16]. (iii) Hybrid P2P energy market: Here, consumers and prosumers exchange energy with one another and with the grid also without relying on a third party. This local energy market can be implemented using the P2P mechanism. This P2P approach manages the market’s local trading and manages the prosumer to grid energy transfers [17]. (iv) Localized P2P market: It is designed for Plug-in Hybrid Electric Vehicle (PHEVs) and does not rely on a trusted third party. In this concept, an interactive double auction process for charging and discharging PHEVs is designed to improve societal welfare. It also offers great security and privacy protection [18]. Then, there is the micro-market, which maintains competition among utility providers for social welfare and ensures network restrictions [11].

In SG, decentralized energy could form a TEM system and revolutionize the marketplace. Several research works have been conducted so far for P2P energy trading, but they have not been explored fully [11,12,13]. The existing infrastructure accomplished a centralized platform (easy to deploy, design, and control) for P2P energy trading to satisfy the energy needs of consumers [19,20]. It also minimizes duplicacy, is easy to access, and is cost-effective [21,22]. However, when trading requests and consumer energy demands rise, the typical centralized SG systems performance (computation and communication costs), delay, network bandwidth, and dependability deteriorate [2]. Many other issues, for instance, single-point-failure, trust, privacy, and security issues also exist [23,24].

**Figure 1:**
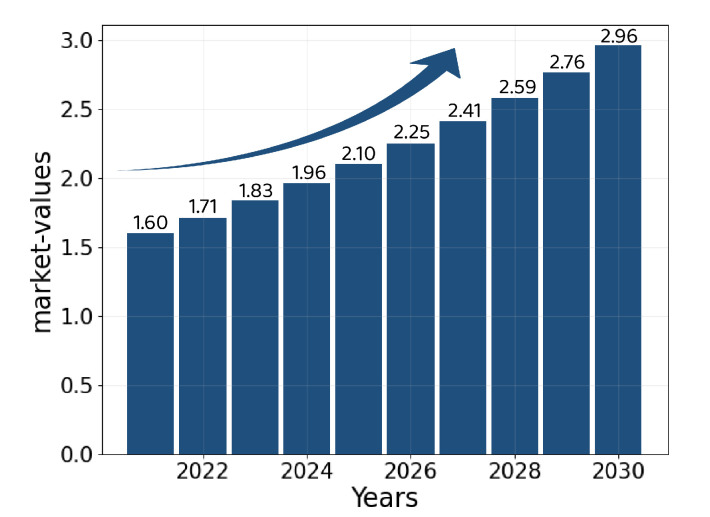
Blockchain-based cryptocurrency market value.

Motivated by the aforementioned gap, this paper proposes the *DT-P2PET* scheme, which is a blockchain-based Decentralized and Transparent P2P Energy Trading scheme. Blockchain is a digital ledge technique that has proven its effectiveness in various applications such as healthcare and cryptocurrencies. Figure 1 represents the growth of the cryptocurrencies market based on blockchain technology, which is growing exponentially and will reach USD 2.96 million by 2030 [25]. The existing blockchain-based energy trading system stores the energy transactions on the blockchain inefficiently, as the cost of storing data on it is high. However, *DT-P2PET* incorporates the InterPlanetary File System (IPFS) mechanism (off-chain storage system) to store data in a decentralized way that resulted in higher throughput [21,26,27,28]. Furthermore, *DT-P2PET* can design Smart Contracts (SCs) in Solidity using a Remix Integrated Development Environment (IDE) with different roles and permissions to all stakeholders of the P2P trading such as prosumers, consumers, and SG administrators.

In *DT-P2PET*, stakeholders are registered on the Ethereum blockchain through the Ethereum client and communicate remotely using 5G-enabled Tactile Internet (TI) to provide high bandwidth during communication and handle data redundancy in the Ethereum-blockchain network [29,30,31]. Next, based on the P2P trading results, *DT-P2PET* proposes a recommender system to increase energy generation through SPV (by increasing the number of prosumers) in the nearby locality of a particular region to reduce the demand–response gap.

### 1.1. Research Contributions

The following are the research contributions of this paper.

- In this paper, we have proposed a blockchain-based decentralized and transparent P2P energy trading scheme, i.e., *DT-P2PET*.
- SCs are designed to minimize the energy generation burden on the grid and benefit consumers as well as prosumers through profit generation in real time.
- The performance of the proposed *DT-P2PET* scheme is evaluated in contrast with existing approaches based on different evaluation metrics such as profit generation and low bandwidth utilization. Next, data storage cost is also reduced using IPFS (an off-chain storage system) with Ethereum blockchain.

### 1.2. Organization of the Paper

The rest of the paper is structured as follows. First, Section 2 narrates the existing works on P2P energy trading and presents a comparative analysis of the proposed *DT-P2PET* scheme with state-of-the-art blockchain-based P2P approaches. Then, the system model and problem formulation of the proposed *DT-P2PET* scheme is discussed in Section 3. Next, Section 4 highlights the workflow of the proposed *DT-P2PET* scheme. Furthermore, the performance evaluation of *DT-P2PET* is discussed in detail in Section 5, and Section 6 concludes the entire research work with future advancements.

## 2. Related Work

Recently, distributed P2P energy trading has been investigated at the microgrid level. Liu et al. [24] proposed a P2P paradigm for neighboring microgrids to save the energy bill and improve DER utilization. Next, Long et al. [11] proposed a P2P trading system and used a game-theoretic approach for the decision-making process. The strategy accomplishes both the optimality and fairness in P2P energy trading, but it necessitates batteries. Dorri et al. [12] demonstrated the Secure Private Energy Trading (SPB) platform as a Proof of Concept (PoC) by using blockchain. For energy trading, SPB minimizes the cost and processing time. However, it lacks scalability by using a bigger testbed in which various energy consumers and providers negotiate and trade energy.

Seven et al. [13] introduced a SC-enabled public Ethereum network for P2P trading. To enable blockchain-based energy trading, the authors proposed an auction-based bidding platform that connects multiple technologies, such as Solidity, Remix, Metamask, Infuro.io, and Ropsten. Then, Han et al. [32] introduced a novel blockchain platform framework to connect producers’ resources with consumers’ needs. Here, more than twenty-five people can trade energy simultaneously using the SC. However, it does not consider expanding the platform’s capabilities, which leads to scalability issues. In light of the blockchain trilemma’s characteristics, Wongthongtham et al. [33] investigated the most effective application of blockchain for P2P energy trading. The result shows that the method is cost-effective.

Moreover, Li He et al. [34] proposed an energy pawn (EP)-based energy sharing structure in a community market with prosumers and consumers. It increases the revenue generated by the EP. Here, customized dynamic pricing and forecasting-based energy capacity scheduling mechanism are presented. However, it lacks the implementation for more accurate energy forecasting, which would improve market efficiency even more. Mehdinejad et al. [35] have proposed blockchain technology-based mechanism for P2P energy token trading. The proposed approach ensures a worldwide and realistic solution while demanding no personal information from consumers and prosumers. However, it lacks implementation in real-life applications. Several works exist for P2P energy trading, but still, it has not been explored fully [7,36]. So, we propose the *DT-P2PET* scheme, which is a blockchain-based decentralized and transparent P2P energy trading scheme.

In the proposed *DT-P2PET* scheme, prosumers and consumers are connected to the Ethereum blockchain and perform P2P energy trading. It helps to maximizes the SG revenue, profits of consumer and prosumer, and reduce burden on the grid. Next, the proposed scheme improves the scalability and latency of the system during trade. Then, an off-chain storage system, i.e., IPFS, is incorporated with the Ethereum blockchain that reduces energy data storage cost. More, Table 1 presents a comparative analysis of the proposed *DT-P2PET* scheme and existing approaches.

## 3. System Model and Problem Formulation

This section presents the system model of the proposed *DT-P2PET* scheme with problem formulation of P2P energy trading in the SG system.

### 3.1. System Model

**Figure 2:**
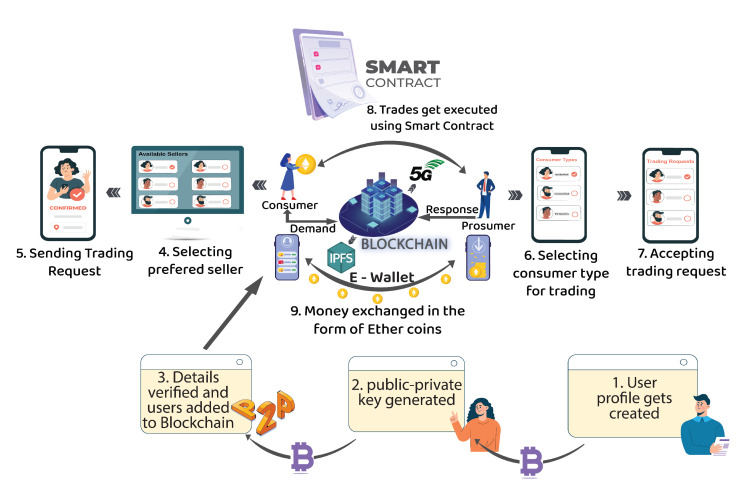
System model of the proposed *DT-P2PET* scheme.

Figure 2 shows the *DT-P2PET*’s system model, which comprises several stakeholders such as SG $\left\{S_{SG},S_{consumer},S_{prosumer}\right\}$, prosumers ($S_{prosumer}),$ consumers ($S_{consumer}$), IPFS protocol, wallets, and the communication link between $S_{prosumer}$ and $S_{consumer}$.

In the beginning, all stakeholders, i.e., $\left\{S_{SG},S_{consumer},S_{prosumer}\right\}$ are registered on the Ethereum blockchain for the community-based transactive energy market. Wallet addresses with public and private keys are created; the consumer’s wallet balance is denoted by $W_{C}$, and for prosumers, it is denoted by $W_{P}$. Then, a prosumer generates energy using SPV (at their place) and first consumes energy by himself ($\xi_{n,c}$). Then, the prosumer initiates a trading request to sell excessive energy in the transactive energy market through a decentralized Ethereum-blockchain platform using SC. The SG administrator verifies the request; then, the amount of energy needed to be traded is published on the blockchain.

The published information on energy is accessible by all buyers (i.e., consumers/prosumers/grid). The prosumers select any type of consumer (residential houses, commercial buildings, EV owners, etc.) based on the shortest distance and minimum battery charging time. The proposed *DT-P2PET* scheme incorporates a user interface and decentralized apps that engage all stakeholders with the SC. The *DT-P2PET* scheme publishes trading information and maintains different information of prosumer and consumer, location details, consumers’ energy requirement, and prosumers’ energy capacity to transact. Here, three different scenarios are captured for trading: (i) prosumers to consumer trading, (ii) prosumers to another prosumer, and (iii) prosumers to grid.

The prosumers-to-consumer trading takes place when excessive energy is required to sell to the needed consumer. Then, prosumer-to-prosumer trading will be executed when a particular prosumer energy demand is higher than the energy he is generating for a particular day. The generated energy is insufficient to meet his own demand; in that scenario, he can use the *DT-P2PET* scheme to purchase energy from another available prosumer. For instance, if the generated energy is 9 kWh and the required energy is 12 kWh, he could purchase 3 kWh from another prosumer using the *DT-P2PET* scheme. Next, the grid can acquire energy through traditional methods or the proposed *DT-P2PET* scheme in case blackout or disruption of energy supply is there. In such a case, the grid will act as a consumer, and trading will be executed between prosumer to the grid using the same approach for energy trading.

As the Ethereum blockchain is not designed to store huge files, hence, we employed the IPFS mechanism in the proposed *DT-P2PET* scheme. To manage the demand of consumers in the TEM system and motivate the prosumer for P2P, a dynamic pricing mechanism is applied based on the mean of the buying and selling price of the energy from the grid. The *DT-P2PET* scheme reduces the energy demand of consumers from the grid and allows them to voluntarily reduce their energy usage (from the grid) during peak hours [39]. The proposed scheme designed a unique SC for this P2P mechanism. When SC requirements are met, the energy transaction is completed; otherwise, the transaction is reverted, and an error notice is published to the Ethereum blockchain.

Once the P2P trading request is approved, the public key is generated for both traders (consumer/prosumer). The private key is generated from the public key, and the user’s wallet address is created using both keys. Wallet addresses are exchanged by the prosumer and consumer so that consumers can pay for the transacted energy. Here, a wallet must contain a sufficient amount of Ether (i.e., Ethereum cryptocurrency), $W_{C}>\alpha$. Here, α is the actual amount to be paid by the consumer to the prosumer. Next, the consumer has to pay the amount α in the prosumer’s wallet $W_{P}$. The wallet’s balance is updated according to the *DT-P2PET* scheme, which is as follows:

$$W_{P}=W_{P}+\alpha$$

(1) $$W_{C}=W_{C}-\alpha$$

### 3.2. Problem Formulation

In the proposed *DT-P2PET* scheme, P2P energy trading guarantees energy transaction security using a distributed ledger system Ethereum blockchain that can handle various issues of centralized systems such as single-point-failure, security, privacy issues, etc. The *DT-P2PET* scheme comprises peers in a decentralized database, each with a digital ledger, i.e., Ethereum blockchain. A consensus algorithm is used by the network to validate each energy transaction between two peers participating in P2P. Energy (*E*) is traded between consumers and prosumers for generated energy ($E_{prosumers}^{G}$) through SPV using price ($E_{price}$) and also emphasis on the distance (dist) between consumers (buyers) and prosumers (sellers). The objectives of the proposed *DT-P2PET* scheme are to minimize the energy generation and supply (χ) burden on the grid. Next, it intends to increase the profit (profit) of both consumers and prosumers using a dynamic pricing mechanism.

Therefore, the objective of the proposed *DT-P2PET* scheme can be denoted as follows.

(2) $$\begin{array}{c} \begin{array}{c} \Theta =min(\chi )+max(profit) \end{array} \end{array}$$

Subject to the following constraints.

$$\begin{array}{c} C1:E_{consumer}>\phi \;\;\;\;\;\;\;\;\;\\ C2:E_{prosumers}^{G}>\phi \;\;\;\;\;\;\;\;\;\\ C3:E_{price}\neq 0\;\;\;\;\;\;\;\;\;\;\;\;\;\;\;\\ C4:\chi \in \{finite,\leq E_{thres}\} \end{array}$$

Next, a prosumer publishes energy data ($\xi_{n,p}>0$) on the Ethereum blockchain for trading; then, the difference between revenues and expenditure is considered as the monetary advantage (φ) to the group of prosumers, which is as follows

(3) $$\begin{array}{c} \varphi =\omega_{g,p}\left[E_{d}\right]-\omega_{g,c}\left[E_{s}\right] \end{array}$$

Here, $E_{s}$ and $E_{p}$ show the energy supply and energy demand with respect to prosumers and consumers. Then, the energy buying price by the grid is $\omega_{g,p}$, and the selling price by the grid is $\omega_{g,c}$. Next, $\omega_{g,p}>\omega_{g,c}$ as merchants (utility supplier) pay prosumers less for their surplus generation since it is considered as non-executable. Hence, a P2P trading price may benefit prosumers while transacting energy with the consumer instead of the grid. So, to achieve it, the proposed *DT-P2PET* scheme uses a mid-market rate for the pricing mechanism for P2P energy trading. Abbreviation Table shows all abbreviations used in this paper.

## 4. The Proposed Approach: *DT-P2PET*

**Figure 3:**
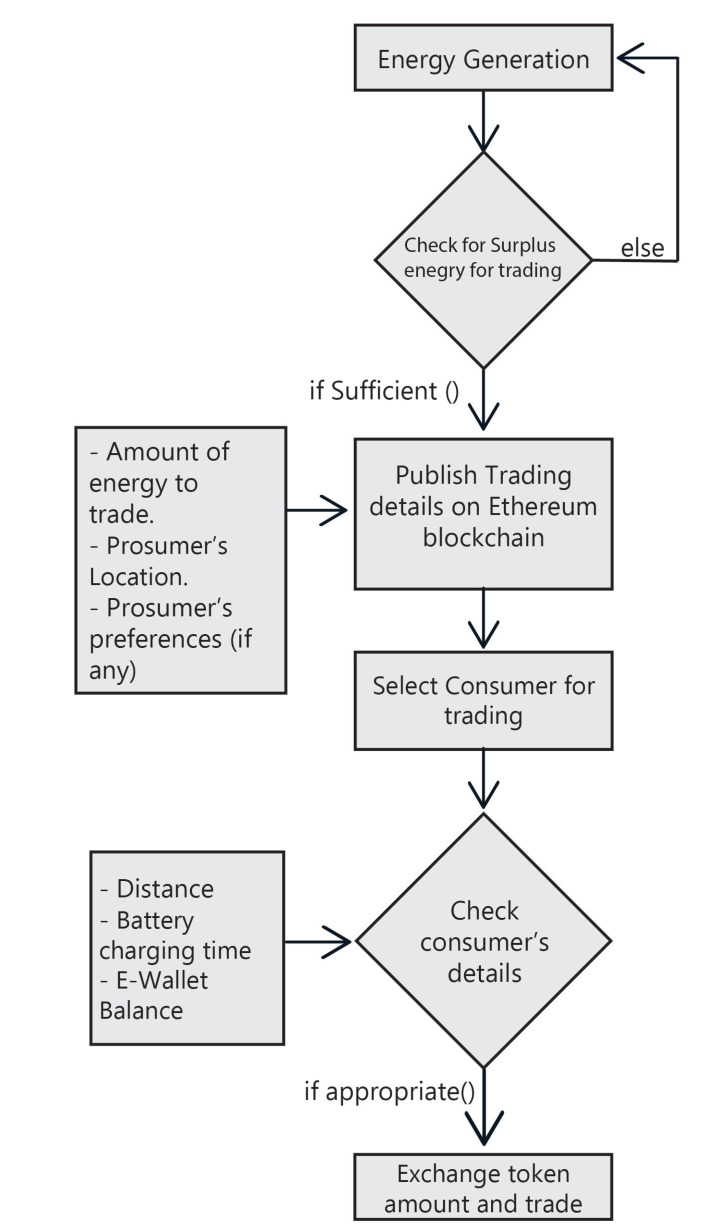
The workflow of the proposed *DT-P2PET* scheme.

Figure 3 represent the workflow of the proposed *DT-P2PET* scheme, which is divided into three phases: (i) Energy Generation, (ii) Energy Data Publishing, and (iii) Energy Trading using P2P. The first phase comprises energy generation, the second phase is data publishing over the Ethereum blockchain for trading, and the third phase is mainly composed of P2P energy trading.

### 4.1. Energy Generation

The first and most important step is to generate energy to consume and trade. The solar panels on the prosumer’s rooftop will generate energy for the prosumer. Prosumers may also use other RES to generate energy. Several factors can determine the amount of energy produced using solar panels [40,41] such as SPV capacity, time of the day, weather, and the location of the SPV. To generate a high amount of energy, the prosumer needs to install a higher capacity SPV. Following the energy generation, the prosumer must decide how much energy to consume and how much energy to use for P2P trading. The more energy used for trading, the more profit the prosumer will receive. If the prosumer lacks sufficient energy for trading, then the prosumer needs to generate the lacking energy for trading.

A prosumer can calculate the energy generation with the help of the following equation:

(4) $$\begin{array}{c} SP_{kW}\times S_{H}\times C=D_{kWh} \end{array}$$

where $SP_{kW}$ represents the prosumer’s SPV capacity (in kWh), $S_{H}$ represents the average number of sunlight hours received by SPV each day, C is the system-dependent parameter ranges between 0 and 1, and $D_{KWh}$ represents the prosumer’s daily energy generation (in kWh). Considering that the prosumer owns 2.5 KW of SPV and receives 6 h of sunlight each day on average, the prosumer’s daily energy generation would be 2.5 × 6 × 0.75 = 11.25 kWh. Here, $D_{kWh}$ is the summation of $\xi_{n,c}$ and $\xi_{n,s}$.

### 4.2. Energy Data Publishing

After the generation of energy, the prosumer must publish details concerning surplus energy to perform trading. Other information about the prosumer, such as location, transaction history, etc. are retrieved from IPFS. A decentralized storage system can be utilized by integrating IPFS with the Ethereum blockchain. IPFS is a peer-to-peer file-sharing network that employs the Merkle direct acyclic graph data structure. It handles data de-duplication and speeds up the energy data-storing process. It hashes the energy data files that are uploaded and then allows users to search for files using the hashes. The proposed scheme uses IPFS to store data files and only broadcasts hashes on the Ethereum blockchain. Algorithm 1 shows the detailed steps for publishing energy data on the Ethereum blockchain and P2P trading.

The reason to use IPFS instead of the Ethereum blockchain for data storage is due to the high data storage cost of Ethereum, which is equivalent to 17,100 USD for 1MB of data. A word in the Ethereum blockchain comprises 256 bits, and the Ethereum gas required to write a word on the Ethereum blockchain is 20,000 gas.

(5) $$\begin{array}{c} 1\;\mathrm{KB}=\frac{2^{10}}{2^{5}}\times 2000\;\mathrm{gas} \end{array}$$

Let G be the current gas price and U be the current price of Ether (Ethereum’s cryptocurrency). Thus, the total cost for ${}^{\prime }x^{\prime }$ words storage can be calculated as follows:

(6) $$\begin{array}{c} T=(G\times U\times \frac{\left(x\times 20,000\right)}{1,000,000,000})USD \end{array}$$

### 4.3. Energy Trading Using P2P

Once the energy data are published over the Ethereum blockchain, the prosumer must select a consumer from among the requested consumers with whom to trade energy depending on certain criteria, such as distance and amount of energy. These parameters are discussed as follows.

#### 4.3.1. Distance

The prosumer must consider the distance (min(dist.)) between his/her place to the consumer’s place with whom the prosumer wishes to make a deal. The buyer (consumer/ another prosumer) must charge their batteries at the prosumer’s location, so distance is indeed a concern. As a result, both the consumer (buyer) and the producer (seller) must consider the distance between them due to the hidden traveling cost. If the distance between them is greater, the consumer’s travel costs will rise, reducing the profit claimed under the proposed scheme. Hence, the distance parameter is one of the crucial parameters in case of the proposed *DT-P2PET* scheme.
**Algorithm 1** Publishing Energy Data on Ethereum blockchain and P2P Trading.**Input:** Type of prosumer/consumer, location, σ,ϵ
{$\psi_{1},\psi_{2},\psi_{3},\ldots ,\psi_{i}\}\in \psi \to$ denotes the list of prosumers and i denotes the number of prosumers.
{$\theta_{1},\theta_{2},\theta_{3},\ldots ,\theta_{j}\}\in \theta \to$ represents the consumers list and j denotes the number of consumers.
σ→ Purchasing price per unit for electric utility companies from prosumers.
ϵ→Purchasing price per unit for consumers from electric utility companies.
μ→ Distance.
**Output: **α→ Dynamic price used for P2P trading.
1:**procedure**Price_mechanism(ψ,θ)2:    $\alpha =\frac{\sigma +\epsilon }{2}$3:    Update α4:**end procedure**5:peer[eth.address]← type, location6:**if **peer[eth.address] upload for the first time **then**7:    **if** peer[eth.address] is prosumer **then**8:        $\psi_{i}\leftarrow peer\left[eth.address\right]$, i++9:    **else**10:        $\theta_{j}\leftarrow peer\left[eth.address\right]$, j++11:    **end if**12:**else**13:    **if** peer[eth.address] is prosumer **then**14:        Update ψ15:    **else**16:        Update θ17:    **end if**18:**end if**// Procedure for selecting consumer.19:**procedure**CONSUMER( μ)20:    **for** $\theta_{i}$∈ θ **do**21:        **if** μ<Threshold **then**22:           *Y*=peer[eth.address]23:           break24:           **if** Balance[Y] >=α **then**25:               **Trade** with the consumer Y26:           **else**27:               CALL_CONSUMER()28:               Trade_Energy_and_Wallet_Update() using Equation (1).29:           **end if**30:        **end if**31:    **end for**32:**end procedure**


#### 4.3.2. Amount of Energy

Aside from distance, the prosumer must consider that the consumer should have a battery/plug-in point to transfer the energy as well as how long the consumer’s battery takes to charge (i.e., amount of energy). Furthermore, the prosumer prefers a consumer who charges the battery in the shortest amount of time (min(charging time)). The amount of energy is a system-dependent parameter whose upper limit needs to be set by the grid.

If all conditions are met and both parties agree, then SC is executed on the Ethereum blockchain. Next, a token amount is transferred from the consumer’s E-wallet to the prosumer’s E-wallet. SC powers the accounting mechanism in P2P energy trading. The remaining payment will be instantly debited from the consumer’s e-wallet and sent to the prosumer’s e-wallet to ensure transparent transactions. The time-frame is defined as hourly for execution of energy trade for a particular price in the proposed scheme.

Energy trading prices are driven by three scenarios:

- Scenario 1: This scenario describes the situation in which the total available surplus energy equals the demand ($\xi_{n,s}=\xi_{n,d}$). In other words, if prosumers engage in P2P trading, they will avoid having energy exchange with electric utility companies (due to low profit generation). The P2P trade price is calculated as follows:
  (7) $$\begin{array}{c} \Omega_{c}=\Omega_{p}=\frac{\omega_{g,c}+\omega_{g,p}}{2} \end{array}$$
- Scenario 2: In this scenario, the total accessible surplus energy exceeds the total demand ($\xi_{n,s}>\xi_{n,d}$). In other words, by participating in P2P trading, prosumers can not only supply energy to energy-deficient consumers but also trade a portion of energy to the different consumers or the electric utility company. The P2P trading buying price is determined in this scenario as follows.
  (8) $$\begin{array}{c} \Omega_{c}=\frac{\omega_{g,c}+\omega_{g,p}}{2} \end{array}$$
  However, the selling price of energy to the utility company is calculated as follows:
  (9) $$\begin{array}{c} \Omega_{p}=\frac{\Omega_{p}\Sigma_{n\in \eta_{c}}\xi_{n,d}+\omega_{g,c}E_{s}}{\Sigma_{n\in \eta_{p}}\xi_{n,s}}. \end{array}$$
- Scenario 3: Here, the total available surplus energy is less than the total demand ($\xi_{n,s}<\xi_{n,d}$). In other words, if prosumers engage in P2P trading, they will be unable to fulfill the demand of consumers experiencing energy outages, compelling them to acquire extra energy by producing more or asking the consumer to acquire the energy from the utility companies themselves.

To motivate prosumers to participate in large amounts, a recommender system is presented where demanded energy exceeds surplus energy: for example, if a particular region has less surplus energy due to various reasons such as a low number of prosumers, high energy demand, weather conditions, and so on. So, to reduce the demand–supply gap, the recommender system will play a crucial role here to recommend more SPV installations in a particular area to generate a huge amount of energy.

Algorithm 1 shows the details steps for P2P trading, and Algorithm 2 explains the recommender mechanism to boost the number of prosumers in the proposed *DT-P2PET* scheme. The next section discusses the experimental result obtained from the proposed *DT-P2PET* scheme.
**Algorithm 2** Recommender system for SPV installation.$\xi_{1,s},\xi_{2,s},\xi_{3,s},\ldots .,\xi_{n,s}\to$ Surplus energy of respective prosumer
$\xi_{1,d},\xi_{2,d},\xi_{3,d},\ldots .,\xi_{n,d}\to$ energy demand of respective consumer
$a_{1},a_{2},a_{3},\ldots .,a_{N}\to$ represents various areas
D→ Total energy demand of any particular area.
S→ Total surplus energy of any particular area.
1:To calculate total energy demand and total surplus energy of any particular area.2:**for**i←1 to *N* **do**3:    **for** j←1 to *n* **do**4:        **if** location of $\xi_{j,d}$ = $a_{i}$ **then**5:           $D\left[i\right]=\xi_{j,d}+D\left[i\right]$6:        **end if**7:        **if** location of $\xi_{j,s}$ = $a_{i}$ **then**8:           $S\left[i\right]=\xi_{j,s}+S\left[i\right]$9:        **end if**10:    **end for**11:**end for**// Check location of consumer and recommend12:**for** i←1 to *N* **do**13:    **if** D[i]>S[i] **then**14:        **for** j←1 to *n* **do**15:           **if** location of $\xi_{j,d}=a_{i}$ **then**16:               Recommend to install SPV to $\xi_{j,d}$ consumer.17:           **end if**18:        **end for**19:    **end if**20:**end for**


## 5. Performance Evaluation

Ethereum is one of the benchmarking tools used for the blockchain network. In this paper, Ethereum SC is designed, and the Mythril tool is used to verify the execution of SC and evaluate the performance of the *DT-P2PET* scheme. Various parameters such as latency, throughput, data storage cost, network bandwidth, etc., are included for evaluation. The system configuration parameters are restricted as per assessment, such as block time, block size, channel, endorsement policy, ledger database, resource allocation, etc. The *DT-P2PET* scheme is evaluated in the windows PC with the following configurations:

- Intel(R) Core(TM) CPU (Intel Core i7 @ 2.6 GHz);
- 16 GB memory;
- 250 GB SSD;
- 1 Gbit/s network.

The proposed scheme is evaluated using the energy data [42] provided by Open Energy Information (OpenEI). Table 2 shows the simulation parameters that are used for the performance evaluation of the proposed *DT-P2PET* scheme.

### 5.1. Dataset Description

The proposed *DT-P2PET* scheme referred to a standard benchmarked dataset, i.e., the OpenEI dataset, which has open-source real-time energy data (freely available) for energy traders, researchers, technology enthusiasts, and policymakers to extract useful information for effective decision making in SG systems. The dataset comprises hourly consumption data of various home appliances such as AC, Basic Facilities, Light, and Miscellaneous (Misc) (considered for the experiment) for Alaska-based residential houses [42]. Next, it has a time-series attribute that specifies the time and date of energy consumption of home appliances. Then, the data are pre-processed, and total hourly consumption is calculated for further analysis.

### 5.2. Experimental Setup and Tools

The proposed scheme is developed using different frameworks and tools, for instance, Truffle suite, Metamask, Remix-IDE, and Ganache network. These tools help in formulating the *DT-P2PET* scheme and phases of the proposed scheme. Next, communication between each stakeholder of the proposed scheme is enabled by wired or wireless networks. Ethereum SC is designed using solidity programming language along with different user-defined functions for buyer, sellers, and administrators. A few of the implemented functions are *registerMeter()* to fetch the prosumer/consumer identity (ID), *addEnergy()* to add the energy for trading in the Ethereum, *getRole()* to assign a specific role to the prosumer, consumer, and SG administrator. Next, a few functions are associated with the trading request, such as *getSellersIds(), getTradeCount(), getTransactionsCount()*, and many more. The SCs are compiled using Truffle-suite, which is a development and testing environment for blockchain-based applications. Once the SC is compiled, it is tested on the Ganache network. If the SC validates the information of the prosumer, consumer, and SG administrator, the energy data are stored inside the IPFS-based Ethereum blockchain. The IPFS converts the energy trading data into the hashed data that improves the data storage cost and data transfere rate of the proposed scheme. The subsequent subsection discusses the performance of the proposed *DT-P2PET* scheme in terms of profit generation, network bandwidth, data storage cost, and data transfer rate.

### 5.3. Profit Generation

**Figure 4:**
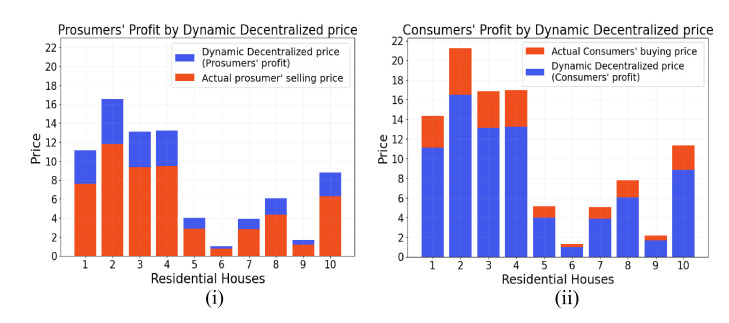
Prosumer/Consumer’s profit using the proposed *DT-P2PET* scheme. (**i**) Prosumer’s profit. (**ii**) Consumer’s profit.

One of the major parameters to motivate prosumers and consumers to participate in the proposed P2P energy trading is profit generation. Therefore, the proposed scheme is designed in such a way that it will generate profit for the consumers as well as prosumers. Figure 4i shows how the prosumer benefits by receiving a higher price for the same amount of energy. Similarly, Figure 4ii illustrates that consumers receive benefits by paying less for the same amount of energy. This happens as the *DT-P2PET* scheme works by taking the mean value of the energy price per unit for buying and selling from the grid, which retains for an hour to perform P2P trading. Consider the case where the prosumer is paid Rs 5 for 1 unit of energy, and the consumer is charged Rs 9 for 1 unit of energy while trading from grid/utility companies. However, in the case of the proposed scheme, the prosumer potentially receives Rs 7 instead of Rs 5 for a single unit, and the consumer has to pay only Rs 7 instead of Rs 9 for a single unit of energy, benefiting both the consumer and the prosumer.

Table 3 illustrates the profit generation for prosumers and consumers using the *DT-P2PET* scheme based on the selling price of 1 kWh as 5 Rs and the buying price of 1 kWh energy for a consumer as 9 Rs for a particular consumer and prosumer. There are three scenarios on which the prosumer’s and consumer’s profit depends, as shown in the previous section. In the first scenario, the total surplus energy with prosumers is equal to the consumer’s demand. As a result, both the prosumer and the customer make identical profits (in Rs). In the second case, the prosumer has more surplus energy to trade than a single consumer would require. As a result, the prosumer will benefit from trading with multiple consumers based on their demand. It can be shown that if a particular prosumer $P_{6}$ has 14 kWh of surplus energy for trading and trades with two consumers, $C_{8}$ and $C_{9}$, he still has 2 kWh of energy remaining. The prosumer now has two options in this case: First, with the electric-utility company, trade the remaining 2 kWh of energy. Second, conserve energy and trade it with other consumers during the subsequent trading session. In the third scenario, the surplus energy available for trade is lower than the demand of a single consumer. As a result, to meet their demand, the consumer will have to trade with more than one prosumer.

### 5.4. Network Bandwidth and Data Storage Cost

Each transaction uploaded to the Ethereum blockchain is relatively expensive; hence, we employed IPFS in parallel with the Ethereum network. IPFS enables high throughput and efficient storage. IPFS yields a hash that is 46 bytes long. As a result, transaction data are stored in IPFS, and IPFS generates a hash for each transaction and is kept in a blockchain block to reduce storage space. The stored transaction can be accessed using the IPFS’s unique hash value, which is known as content-addressable access. The IPFS content-addressed mechanisms denote the significance of the transaction and offer peers confidence that they are accessing the precise information that the prosumer or consumer has saved. Transactions are credible and persistent due to the hash generated through IPFS.

**Figure 5:**
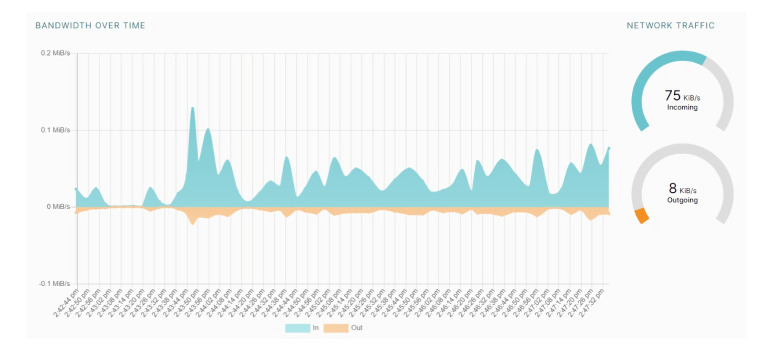
IPFS Network Status.

**Figure 6:**
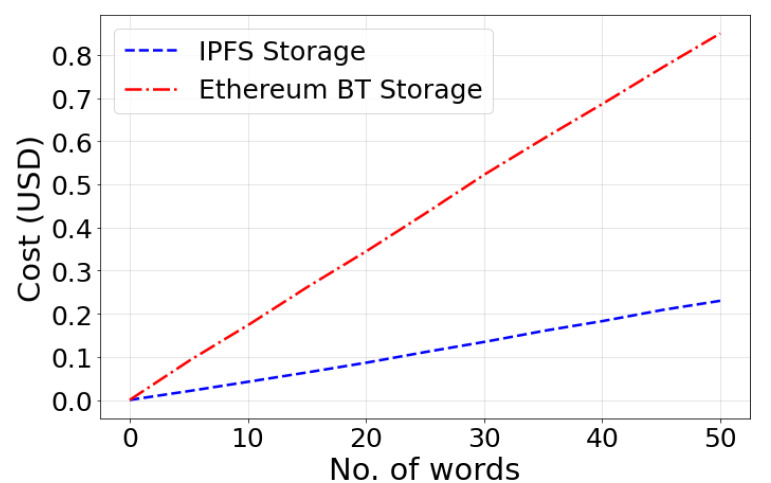
IPFS Storage Cost.

Figure 5 shows the network bandwidth used by the IPFS while performing energy trading. The blue area depicts the network’s download bandwidth, while the orange area depicts the network’s upload bandwidth. Figure 6 depicts the energy trading transactions data storage cost in the proposed *DT-P2PET* scheme using the IPFS mechanism while performing energy trading and comparing the same with existing blockchain-based approaches (not including IPFS). It depicts from the figure that the proposed *DT-P2PET* scheme has low cost (compared to the existing approach) even with the increasing number of words.

### 5.5. Data Transfer Rate

The competition to develop a more sophisticated energy trading mechanism is heating up. However, the blockchain system needs a higher-speed network communications service. Fifth-generation (5G) technology is intended to be completely deployed to deliver more reliable, consistent, and faster connection for communication. To fully utilize the IPFS and blockchain’s capabilities, a 5G technology network is required, as greater bandwidth can be accomplished with the help of 5G technology (a communication platform that can provide incredibly low latency and great reliability). This enables energy transactions to be executed quickly and efficiently.

**Figure 7:**
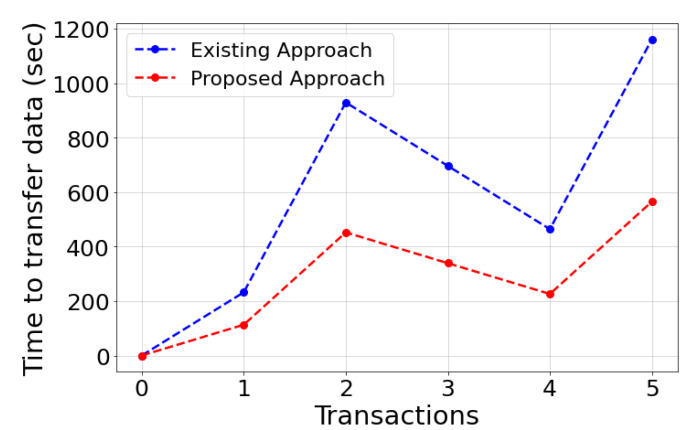
Transfer rate comparison.

Figure 7 compares network statics in the proposed *DT-P2PET* scheme and existing 4G-based approaches. This figure depicts that the proposed approach outperforms the existing approaches in every transaction, regardless of transaction size.

### 5.6. Scalability

**Figure 8:**
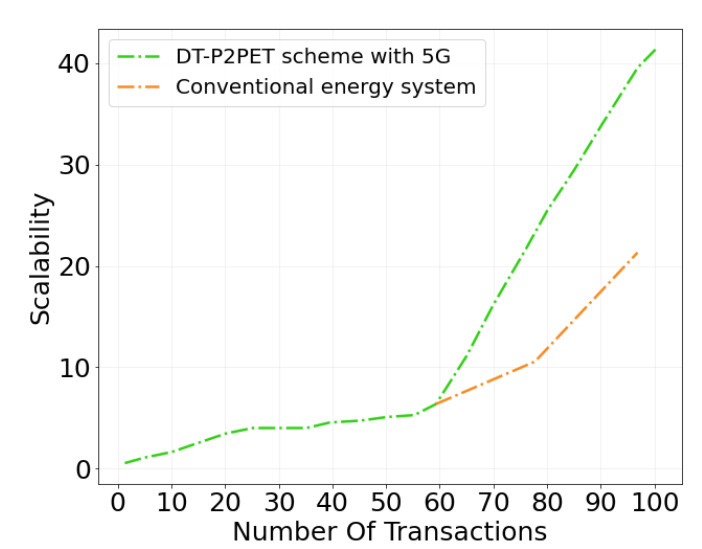
Scalability comparison.

Figure 8 illustrates the system scalability comparison of the proposed *DT-P2PET* scheme with existing 4G-based approaches. Here, the x-axis represents the number of transactions per minute, while the vertical axis represents the number of users that can be increased by the system. When the number of transactions is low, the proposed scheme does not have much impact in terms of scalability; however, as the number of transactions increases, the suggested *DT-P2PET* scheme continues to gain scalability, using the IPFS system and 5G network with Ethereum, but the existing approaches quickly hit bottlenecks.

## 6. Conclusions

In this paper, we proposed a P2P energy trading scheme, i.e., the *DT-P2PET* scheme using the Ethereum blockchain. The *DT-P2PET* scheme is a decentralized, transparent, and secure P2P trading approach with the real-time settlement of trades based on blockchain technology. An Ethereum client is utilized in the *DT-P2PET* scheme, and SCs are employed to carry out the energy trading. The development of SCs facilitated P2P energy trading using a dynamic pricing mechanism. Here, *DT-P2PET* also used the IPFS method for off-chain energy data storage that handles data storage costs and improves the scalability of the system. Furthermore, by employing a dynamic pricing mechanism, *DT-P2PET* strives to benefit both consumers and prosumers. The *DT-P2PET* technique is developed and deployed using the Truffle suite, and block verification is completed in Remix IDE. The performance of the proposed *DT-P2PET* scheme is evaluated by comparing it to existing blockchain-based techniques in terms of data storage cost, scalability and data transfer rate. The experimental result proved the effectiveness of the *DT-P2PET* scheme.

In the future, we will verify user latency and privacy in the proposed scheme on different blockchain platforms as an extension of this research work. Next, the proposed P2P energy trading scheme for different load profiles will be studied in depth in the near future.

## Figures and Tables

**Table 1:** A comparative analysis of the proposed *DT-P2PET* scheme with the existing approaches.

Approaches	Year	Short Description	Merits	Demerits
Long et al. [11]	2018	A game-theoretic-based framework for energy trading and decision-making process	The approach achieves both optimality and fairness for P2P trading	The model works on batteries only
Morstyn et al. [37]	2019	A P2P energy-trading contract networks, which is bilateral and scalable	Emphasizes trading strategies for real-time and forward contracts	Need to be implement for practical aspect
Musleh et al. [38]	2019	It examined emerging blockchain applications and its utilization in the SG	It demonstrated benefits of blockchain in the electrical network and SG framework	Scalability and latency issue
Dorri et al. [12]	2019	Presented a Secure Private Blockchain-based (SPB) energy trade architecture as a PoC	SPB minimizes energy trading costs, blockchain size, and processing time.	It lacks the scalability, and SPB implementation requires a bigger testbed
Seven et al. [13]	2019	A public and SC-enabled auction-based bidding platform is proposed for energy trading	Auction-based energy-trading platform	Need to improve optimal and efficient functioning of the proposed approach
Han et al. [32]	2020	A blockchain-based framework to bridge the demand–response gap of producers’ energy production and consumers’ needs in P2P energy trading	It allows more than 25 users to trade energy at the same time	Lacks the consideration of enriching the functions of the platform and testing the same in different environments
Wongthongtham et al. [33]	2021	Examines the most effective use of blockchain technology for P2P energy trading	The approach is cost effective for blockchain transaction	Data storage cost issue on blockchain and scalability issue
L He et al. [34]	2021	In a community market with a shareholder energy storage system, prosumers, and consumers; it suggested an energy-sharing framework which is based on energy pawn (EP)	Maximizes the energy pawn’s revenue generation	For efficient outcomes, netload forecasting must be improved
Mehdinejad et al. [35]	2022	Leverage blockchain platform to achieve P2P energy token trading	The proposed approach ensures a worldwide and realistic solution while demanding no personal information from the participants	The efficiency of the approach needs to be improved
The proposed *DT-P2PET* scheme	2022	A blockchain-based decentralized and transparent P2P energy-trading scheme	Maximizes the SG revenue, profits of consumer and prosumer, reduces burden on grid	-

**Table 2:** Simulation Parameters Setting.

Parameters	Configuration
Transactions	1000 per round
Rounds	5
Transactions mode	Read/Write
Rate	50 to 250 tps
Varied factor	Block size with transaction rate

**Table 3:** Profit generation for prosumer and consumer.

Scenario	Prosumer	Total Production (kWh)	Surplus Energy (kWh)	Profit (Rs)	Consumer	Total Demand (kWh)	Profit (Rs)
	P1	11	5	10	C1	5	10
$\xi_{n,s}=\xi_{n,d}$	P2	15	9	18	C2	9	18
	P3	23	12	24	C3	12	24
	P4	11	8	16	C4	5	10
					C5	3	6
$\xi_{n,s}>\xi_{n,d}$	P5	15	10	20	C6	6	12
					C7	4	8
	P6	23	14	24	C8	2	24
					C9	10	20
	P7	12	7	14	C10	14	28
	P8	10	7	14			
$\xi_{n,s}<\xi_{n,d}$	P9	15	6	12	C11	10	20
	P10	18	5	8			
	P11	9	4	8	C12	9	18
	P12	23	7	10			

## Data Availability

Not applicable.

## Abbreviations

The following abbreviations are used in this manuscript:

**Symbols**	**Descriptions**
$\xi_{n,s}$	Surplus energy of prosumer
$\xi_{n,d}$	Energy demand of consumer
$E_{s}$	After trade, the total surplus energy in SG
$E_{d}$	After trade, total excess energy demand in SG
φ	Coalition
$\eta_{p}$	Set of prosumers
$\eta_{c}$	Set of consumers
$\Omega_{p}$	P2P selling price
$\Omega_{c}$	P2P buying price
$\omega_{g,c}$	Buying prices by the grid
$\omega_{g,p}$	Selling prices by the grid
$\xi_{n,c}$	Energy consumed by the prosumer itself
